# Using conversation analytic methods to explore the content, delivery and receipt of complex healthcare interventions

**DOI:** 10.1186/1745-6215-16-S2-P77

**Published:** 2015-11-16

**Authors:** Rebecca K Barnes, Marcus Jepson, Alison Heawood

**Affiliations:** 1 University of Bristol, Bristol, UK

## 

The majority of healthcare interventions rely on communication of one kind or another. In some trials, the main intervention elements are communication-based, involving multiple parties. However there has been little research on the content, delivery or receipt, or on the communication outcomes (i.e. the interactional consequences) of such complex interventions.

In this paper we demonstrate the benefits of such an approach in three separate case studies where conversation analytic (CA) methods have been used in primary care trials. CA is a well-established qualitative method that affords opportunities for the identification, and distributional analyses, of communication practices of interest, providing evidence for the interactional consequences of communication-based interventions.

Case study 1 explored interactions between patients and practitioners in a follow-up study to a trial of online cognitive behavioural therapy for depression. Case study 2 compared interaction in nurse-led vs GP-led telephone triage for same-day appointment requests in a sub-study to a trial evaluating effectiveness and cost effectiveness. Case study 3, explored GP’s fidelity to communication training in a feasibility study of a consultation-level intervention for the management of frequent attenders with clinically inexplicable symptoms.

Understanding how both patient and practitioner communication behaviours can influence the content, delivery and receipt of an intervention is important. Without an understanding of how communication can be consequential, we are left with idealisations about how an intervention should work, drawing on formal theories alone, as opposed to opening a window on the similarities, gaps or differences between an intervention-as-designed and how it plays out in practice.

